# Outcomes and Risk Factors in Young Patients with Head-and-Neck Cancer: A Multi-Center Retrospective Analysis

**DOI:** 10.3390/medicina62071383

**Published:** 2026-07-17

**Authors:** Fabian Baier, Leila Erpenstein, Julia Maurer, Karolina Mueller, Felix Steger, Isabella Gruber, Julian Kuenzel, Matthias Hautmann, Oliver Koelbl, Christoph Suess

**Affiliations:** 1 Klinik und Poliklinik für Strahlentherapie, Universitätsklinikum Regensburg, 93053 Regensburg, Germanyc.suess@ukr.de (C.S.); 2 UCC-R Universitäres Onkologisches Zentrum Regensburg, 93053 Regensburg, Germany; 3 Zentrum für Klinische Studien, Universitätsklinikum Regensburg, 93053 Regensburg, Germany; 4 Klinik und Poliklinik für Hals-Nasen-Ohren-Heilkunde, Universitätsklinikum Regensburg, 93053 Regensburg, Germany; 5 Klinik für Strahlentherapie und Radioonkologie, Klinikum Traunstein, 83278 Traunstein, Germany

**Keywords:** Head-and-neck cancer, radiotherapy, chemotherapy, young patients, risk factors

## Abstract

*Background and Objectives:* Head and neck cancer in patients aged 40 years or younger represents a rare and heterogeneous entity with conflicting data regarding prognosis and risk factors. This study aimed to evaluate oncological outcomes and independent prognostic factors in young patients treated with radio(chemo)therapy. *Materials and Methods*: This retrospective multi-center analysis included patients aged ≤ 40 years with histologically confirmed HNC who received definitive or adjuvant radio(chemo)therapy between 2002 and 2023 at the University Hospital Regensburg and affiliated partner hospitals. Overall survival (OS) and progression-free survival (PFS) were estimated using the Kaplan–Meier method. Independent prognostic factors were identified by Cox proportional hazard regression with backward stepwise elimination. *Results:* A total of 88 patients were included. The median age at diagnosis was 38.2 years (IQR 35.8–39.8). Median OS was 91.0 months in the adjuvant and 23.7 months in the definitive treatment group. In multivariate analysis, four independent predictors of OS were identified: nicotine abuse (HR 2.887; *p* = 0.004), pre-existing comorbidities (HR 2.871; *p* = 0.005), absence of complete remission 12 weeks after radiotherapy (HR 25.676; *p* < 0.001), and locoregional recurrence or distant metastases (HR 8.183; *p* < 0.001). Failure to achieve complete remission was the sole independent predictor of PFS (HR 4.479; *p* < 0.001). *Conclusions:* In young HNC patients, early treatment response and disease recurrence are the strongest determinants of survival, alongside modifiable lifestyle factors and comorbidity burden. These findings support the need for intensified response monitoring and tailored follow-up strategies in this patient population.

## 1. Introduction

In 2023, 4230 women and 8650 men were diagnosed with head and neck cancer (HNC) in Germany, and 1428 women and 3768 men died from the disease in the same year. Approximately 85% of head and neck cancers are squamous cell carcinomas. While the incidence among women has remained largely stable since 2011, a slight decline has been observed in men over the same period. The median age at diagnosis in Germany is 68 years for women and 66 years for men [1]. Consistent with this age distribution, the incidence of head and neck cancer is significantly lower in patients younger than 50 years compared with those aged 50–69 years, with reported rates of 3 versus 26.9 cases per 100,000, respectively [2].

Defining a distinct cohort of “young patients” remains challenging, as no universally accepted age cutoff exists; however, previous studies have frequently applied an upper age limit ranging from 40 to 45 years [3,4,5,6,7,8,9,10,11,12,13]. Data regarding clinical outcomes in this younger population are conflicting, with some studies reporting comparable or improved overall and disease-free survival [4,5,6,7,13]. Notably, a meta-analysis by Panda et al. indicates that younger patients may experience a significantly higher recurrence rate and worse disease-free survival [14]. These findings have fueled ongoing debate as to whether differences in outcomes reflect distinct tumor biology and, potentially, altered disease behavior in younger patients with head and neck cancer [15,16,17,18,19,20,21,22]. Accordingly, Frei et al. have proposed investigating intensified treatment protocols for this patient population [3].

The primary objective of this retrospective multi-center analysis was to evaluate oncological outcomes—specifically overall survival and recurrence-free survival—in patients aged ≤ 40 years with head and neck cancer treated with definitive or adjuvant radio(chemo)therapy. The secondary objectives were to identify independent prognostic factors associated with these endpoints and to compare survival outcomes between the definitive and adjuvant treatment groups. Better characterization of high-risk subgroups within this population may clarify the heterogeneity observed across prior studies and enable more refined risk stratification. Ultimately, improved identification of vulnerable patient cohorts could support the development of individualized surveillance strategies and targeted treatment approaches.

## 2. Materials and Methods

### 2.1. Study Design and Patient Characteristics

This retrospective multi-center study analyzed patients with histologically confirmed head and neck cancer who received definitive or adjuvant radio(chemo)therapy (R(C)T) between 2002 and 2023. Inclusion criteria were defined as follows: (1) age between 18 and 40 years at the time of initial diagnosis; (2) histologically confirmed malignancy of the head and neck region, encompassing the following tumor entities: oropharynx, oral cavity (oral tongue, floor of mouth, gingiva, buccal mucosa, hard palate), hypopharynx, larynx, nasopharynx, nose and paranasal sinuses, salivary glands, and cancer of unknown primary (CUP); (3) treatment with definitive or adjuvant radio(chemo)therapy; and (4) discussion of the individual case in the interdisciplinary head and neck tumor board at the University Hospital Regensburg prior to treatment allocation. Patients were treated at the University Hospital Regensburg or at affiliated partner hospitals within the Comprehensive Cancer Center Ostbayern (see Acknowledgments); all surgical procedures were performed at the University Hospital Regensburg. Exclusion criteria were (1) age below 18 years or above 40 years at the time of diagnosis; (2) histological diagnosis of thyroid carcinoma, lip carcinoma, or cutaneous malignancy, which were excluded due to their distinct etiology, biological behavior, and treatment approach compared to mucosal head and neck carcinomas; (3) patient objection to the use of clinical data for research purposes.

The age cutoff of 40 years was deliberately selected as the lower end of thresholds commonly applied in the literature, where cutoffs of 40 to 45 years have been most frequently used [3,4,5,6,7,8,9,10,11,12,13,14]. By applying this more restrictive criterion, the aim was to isolate an especially young patient collective in whom distinct or potentially more aggressive tumor biology may be of particular relevance, thereby allowing a more focused characterization of this understudied subgroup.

The study was conducted in accordance with the Declaration of Helsinki and was approved by the Ethics Committee of the University of Regensburg (protocol code 24-3694-104; date of approval: 25 March 2024).

A total of 88 patients aged ≤ 40 years were included, of whom 64 (72.7%) were male and 24 (27.3%) were female. The median age at initial diagnosis was 38.2 years (22.6–40.9 years, IQR 35.8–39.8 years). The majority of patients presented with advanced disease, with UICC stage IVa being the most frequent stage (n = 57; 64.8%). Tumors were most commonly located in the oropharynx (n = 29; 33.0%) and oral cavity (n = 21; 23.9%). Detailed patient, tumor, treatment, and outcome characteristics are summarized in Table 1. A comprehensive dataset can be found in the Supplementary Material (Table S1). The median follow-up duration was 21.9 months (IQR 9.7–69.0 months).

### 2.2. Data Collection and Follow-Up

Clinical data were extracted from institutional electronic documentation systems, including Mosaiq (IMPAC Medical Systems, Elekta, Stockholm, Sweden), SAP (SAP, Walldorf, Germany), Onkodat (MedicDAT GmbH, Poikam, Germany), and CATO (Becton Dickinson, Franklin Lakes, NJ, USA). Survival status was verified using records from the local registration office and the Tumor Center Regensburg.

Acute and late treatment-related toxicities were documented during therapy and at scheduled follow-up visits (6 weeks, 3 months, 6 months, and 12 months after completion of radiotherapy). Toxicities were graded according to the Common Terminology Criteria for Adverse Events (CTCAE), version 5.0, consistent with documentation standards applied at the time of treatment [23]. The analysis considered radiation dermatitis, mucositis, xerostomia, trismus, loss of taste, dysphonia, dysphagia, weight loss, and hematologic alterations (leukopenia, anemia, thrombocytopenia).

### 2.3. Statistical Analysis

Statistical analyses were performed using IBM SPSS Statistics, version 25 (IBM, Armonk, NY, USA). A two-sided *p*-value < 0.05 was considered statistically significant. Continuous variables were analyzed using *t*-tests, ordinal variables using Mann–Whitney U tests, and categorical variables using Chi-squared tests.

Overall survival (OS) and progression-free survival (PFS) were estimated using the Kaplan–Meier method with a maximum follow-up of 60 months and compared using the log-rank test. Predictors of treatment feasibility and overall survival were assessed using Cox proportional hazards regression models. Variables with a *p*-value < 0.10 in univariate analyses were entered into multivariate models to identify independent predictors while adjusting for potential confounding factors. Statistical significance in multivariate analyses was defined as *p* < 0.05.

## 3. Results

### 3.1. Treatment Characteristics

**Figure A1:**
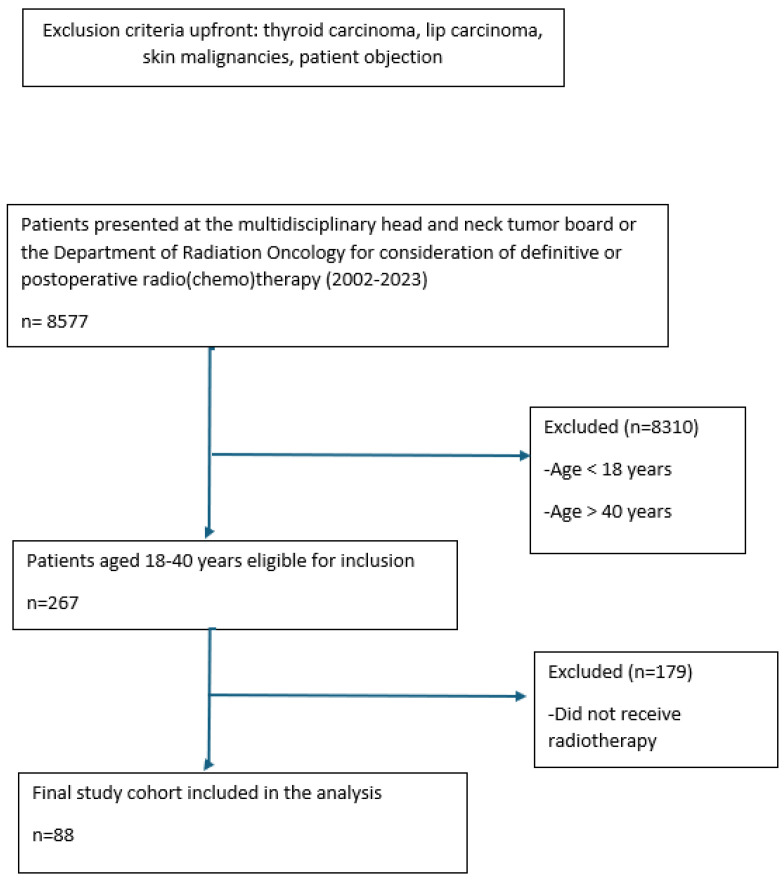
STROBE Diagram of participant flow.

A total of 88 patients aged ≤ 40 years were included (Figure A1). Concurrent platinum-based chemotherapy regimens were used, including cisplatin, carboplatin, or cisplatin combined with 5-fluorouracil. The most commonly applied regimen was weekly cisplatin at a dose of 40 mg/m^2^, corresponding to a planned cumulative dose of 200 mg/m^2^ administered over five cycles.

Combined radiochemotherapy was administered to 59 patients (67.0%). Treatment was delivered in the adjuvant setting in 52 patients (59.1%), while 36 patients (40.9%) received definitive radiotherapy or RCT.

Radiotherapy was delivered using either conventionally fractionated schedules in 89.7% of patients or hyperfractionated accelerated regimens in 10.2% of cases (Table 2). Conventional fractionation was most commonly applied with 1.8–2.0 Gy per fraction up to a cumulative dose of 54.0–72.0 Gy, and hyperfractionated regimens with 1.5–2.0 Gy up to a cumulative dose of 69.6–72.0 Gy. All treatments were performed using linear accelerators. Three-dimensional conformal radiotherapy techniques were employed, most frequently intensity-modulated radiotherapy (IMRT) or volumetric modulated arc therapy (VMAT).

### 3.2. Feasibility of Therapy

R(C)T was generally well tolerated and could be completed as planned in the majority of patients. Of the total cohort, 52 patients (59.1%) received adjuvant R(C)T and 36 (40.9%) received definitive R(C)T. Simultaneous systemic therapy was administered to 59 patients (67.0%). The full initially prescribed radiotherapy dose was delivered to 80 patients (90.9%).

Ongoing radio(chemo)therapy had to be discontinued prematurely in 8 patients (9.1%) due to treatment intolerance, complications, comorbidities, or patient preference. Of the 59 patients who received simultaneous chemotherapy, 38 (64.4%) completed more than 75% of the prescribed chemotherapy dose.

Acute toxicities of at least CTCAE v5 Grade 3 occurred in 30 of 73 evaluable patients (41.1%). Late toxicities, defined as those occurring 90 days or more after the start of radiotherapy, were documented in 40 patients (45.5%).

### 3.3. Nutrition During Radiotherapy

By the end of therapy, 26 patients (29.5%) were able to eat orally, while 53 (60.2%) required parenteral nutrition or feeding tube support during radio(chemo)therapy. Nutritional status at the end of treatment was not documented in 9 patients (10.2%).

Complete weight data were available for 60 patients (68.2%). Of these, 29 patients (48.3%) experienced no weight loss or a weight loss of up to 5 kg at 6 months after completion of therapy compared to baseline. The remaining 31 patients (51.7%) lost more than 5 kg during treatment.

### 3.4. Survival Outcomes and Therapeutic Response

**Figure 1:**
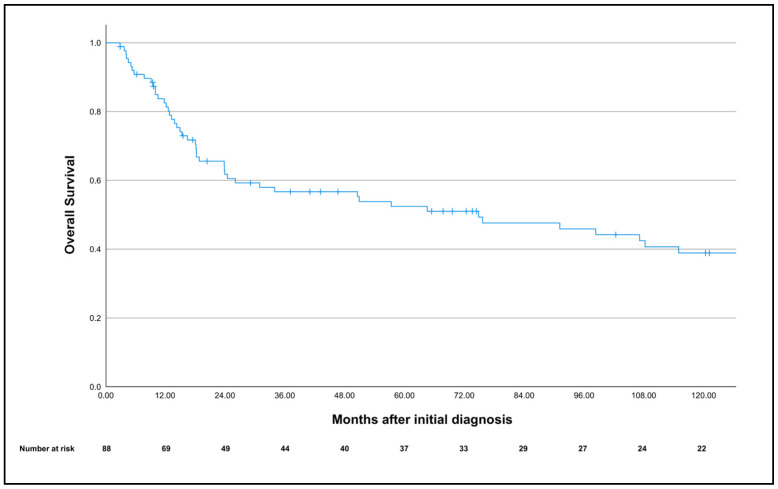
Overall survival of the entire cohort (months).

According to Kaplan–Meier analysis, the overall survival rate for the entire cohort was 82.5% after 12 months, 56.7% after 36 months, and 52.4% after 60 months. The median overall survival was 74.7 months (IQR 26.6–122.8 months, Figure 1).

**Figure 2:**
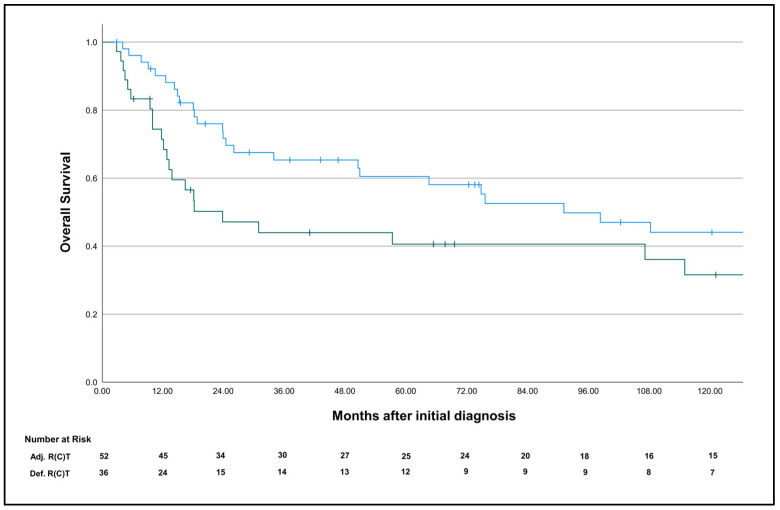
Overall survival: adjuvant (blue) vs. definitive situation (green).

A marked, but not statistically significant, difference in overall survival (*p* = 0.078) was observed when comparing the two therapeutic approaches—adjuvant versus definitive RCT: 90.2% vs. 71.4% at 12 months, 65.4% vs. 44.0% at 36 months and 60.5% vs. 40.6% at 60 months. The median overall survival was 91.0 months (IQR 46.6–135.4 months) vs. 23.7 months (IQR 4.2–43.2 months, Figure 2).

**Figure 3:**
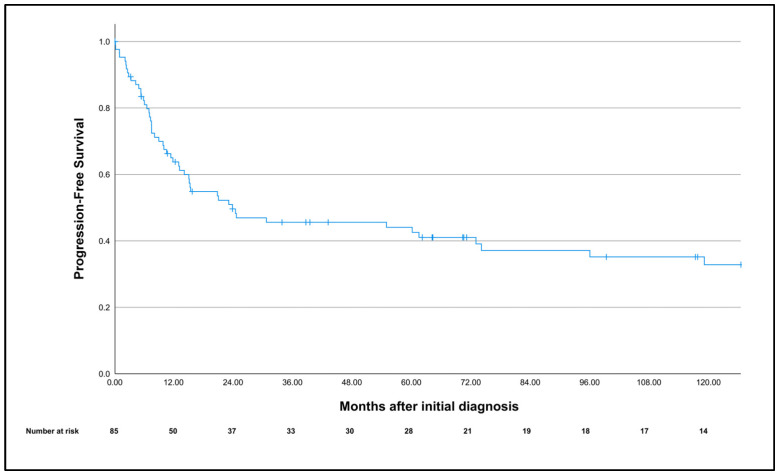
Progression-free survival of the entire cohort (months).

Progression-free survival in the overall cohort was 63.8% at 12 months, 45.6% at 36 months and 42.6% at 60 months. The median progression-free survival in the overall cohort was 23.7 months (IQR 0–59.8 months, Figure 3).

**Figure 4:**
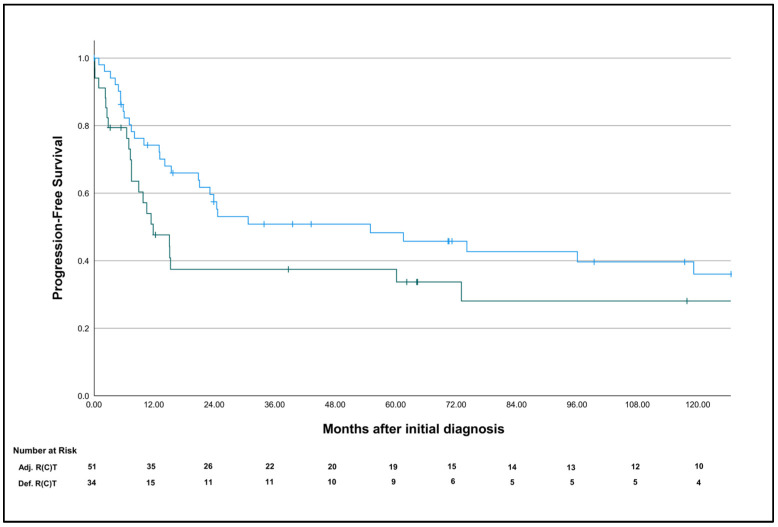
Progression-free survival: adjuvant (blue) vs. definitive situation (green).

Progression-free survival rates were numerically higher in the adjuvant group compared with the definitive group at all time points—74.2% vs. 47.6% at 12 months, 50.8% vs. 37.4% at 36 months, and 48.3% vs. 33.7% at 60 months—although these differences did not reach statistical significance (Figure 4).

Complete remission was achieved in 69.3% of patients at 12 weeks post-treatment. Over the entire observation period, locoregional recurrence and distant metastases were observed in 26.1% and 22.7% of patients, respectively.

### 3.5. Disease Stage Distribution by Treatment Group

UICC stage distribution differed significantly between the two treatment groups. Patients receiving definitive RCT presented with significantly more advanced disease stages compared to those in the adjuvant group (Mann–Whitney-U = 498.500; Z = −3.423; *p* < 0.001; effect size r = −0.380). In the adjuvant group, UICC stage IVa was the most frequent stage (67.3%), with only 2.0% of patients presenting with stage IVb. In contrast, 75.0% of patients in the definitive group presented with stage IVa and 15.6% with stage IVb or IVc.

### 3.6. Univariate and Multivariate Analysis

To identify independent factors influencing overall survival, Cox proportional hazard regression with backward stepwise elimination was performed, proceeding from univariable to multivariable modelling. Of the 88 patients, 71 met eligibility criteria and were included in the analysis. In the final model, four independent predictors of overall survival were identified: nicotine abuse, pre-existing comorbidities, absence of complete remission 12 weeks after radiotherapy, and locoregional recurrence or distant metastases.

Nicotine abuse and pre-existing comorbidities were each significantly associated with increased mortality risk, with comparable effect sizes. Smokers had a 2.887-fold higher risk of death compared with non-smokers (HR = 2.887; 95% CI: 1.408–5.924; *p* = 0.004), and patients with comorbidities had a 2.871-fold higher risk compared with those without (HR = 2.871; 95% CI: 1.372–6.005; *p* = 0.005). Pre-existing comorbidities were documented in 37 patients (42.0%), encompassing a heterogeneous spectrum of conditions; the most frequently recorded were prior malignancies and hepatic disease (liver cirrhosis/fibrosis).

Absence of complete remission 12 weeks after radiotherapy was associated with a markedly increased risk of mortality. Patients with partial remission or progressive disease demonstrated a more than 25-fold higher risk of death compared with those achieving complete remission (HR = 25.676; 95% CI: 9.369–70.365; *p* < 0.001).

Locoregional recurrence or distant metastases were significantly associated with reduced overall survival, with an approximately eightfold higher risk of death in affected patients (HR = 8.183; 95% CI: 3.295–20.315; *p* < 0.001).

To verify the final model, an additional multivariable Cox proportional hazard regression was performed. The results confirmed all four predictors identified through backward elimination, with no clinically relevant deviations in *p*-values, hazard ratios, or confidence intervals, further supporting the stability and robustness of the model: nicotine abuse (HR = 2.891; 95% CI: 1.242–6.727; *p* = 0.014), pre-existing comorbidities (HR = 2.871; 95% CI: 1.474–5.594; *p* = 0.002), absence of complete remission 12 weeks after radiotherapy (HR = 25.676; 95% CI: 9.369–70.365; *p* < 0.001), and locoregional recurrence or distant metastases (HR = 8.183; 95% CI: 3.296–20.315; *p* < 0.001).

An analogous analysis was performed to determine independent predictors of progression-free survival, applying Cox proportional hazard regression with backward stepwise elimination from univariable to multivariable modelling. Of the 88 patients, 62 met eligibility criteria and were included in the analysis.

At the final stage of model selection, one independent predictor was identified. Failure to achieve complete remission 12 weeks after radiotherapy was the only variable with a significant impact on progression-free survival (*p* < 0.001). Patients with partial remission or progressive disease at 12 weeks had more than a fourfold increased risk of an event —defined as death or recurrence—compared with those achieving complete remission (HR = 4.479; 95% CI: 2.240–8.948).

## 4. Discussion

### 4.1. Survival Outcomes and Comparison with the Literature

Comparison of survival outcomes across studies of young HNC patients is inherently limited by methodological and cohort heterogeneity. Beyond differing definitions of the “young” patient population, included tumor subsites, stage distribution, and treatment modality vary substantially across studies. Notably, our cohort applies a particularly stringent age cutoff of 40 years, while comparable contemporary studies defined their young patient populations with an upper age limit of 45 years. Bearing these limitations in mind, the survival outcomes observed in our cohort remain broadly comparable to those reported in contemporary literature on young HNC patients.

Frei et al. [3], who analyzed young HNSCC patients (≤45 years) treated with RCT at two German tertiary centers, reported 2-year and 5-year OS rates of 79.7% and 67.1%, respectively, and 2- and 5-year DFS rates of 73.4% and 67.1%. While these figures exceed those observed in our study (5-year OS 52.4%), they are more closely aligned with our adjuvant cohort (3-year OS 65.4%; 5-year OS 60.5%). This discrepancy can be explained by differences in stage distribution: our cohort included a higher proportion of UICC stage IVa and IVb patients (64.8% and 6.8% respectively), which is substantially greater than in Frei et al. (49.4% stage IVa, 10.1% stage IVb).

A further point of comparison is offered by Fan et al. [11], who retrospectively analyzed 100 young patients (≤45 years) with oral cavity squamous cell carcinoma treated between 2001 and 2010, reporting a 5-year OS of 75.5% and a 5-year DFS of 61.0% with a median follow-up of 68.8 months. When comparing those figures, two key distinctions have to be considered. Fan et al. exclusively focused on oral cavity, which excluded the more advanced oropharyngeal and other subsites represented in our cohort, and the surgical basis of their treatment approach, with all patients undergoing primary resection.

Similarly, Zou et al. [4], reporting on 99 young HNSCC patients (≤45 years) from two large German radiation oncology centers, observed a median OS of 63 months with 1-year, 3-year, and 5-year OS rates of 93%, 69%, and 57%, respectively, and 1-year, 3-year, and 5-year PFS rates of 78%, 53%, and 47%. While the median OS in our study was numerically higher at 74.7 months, the more favorable 5-year OS reported by Zou et al. compared to our overall cohort is similar to Frei et al., attributable to the lower proportion of UICC stage IV patients as well as the higher proportion of surgically treated patients in their study (70.7% vs. 59.1% in our cohort). Notably, the inferior survival outcomes observed in our definitive treatment subgroup compared to the adjuvant group (median OS 23.7 vs. 91.0 months) should be interpreted in the context of a significantly more advanced UICC stage distribution in the definitive cohort (Mann–Whitney-U = 498.500; Z = −3.423; *p* < 0.001; effect size r = −0.380), with 75.0% of definitive patients presenting at UICC stage IVa and 15.6% at stage IVb or IVc, compared to 67.3% and 2.0%, respectively, in the adjuvant group. To our knowledge, ours is the first study to formally demonstrate a statistically significant difference in UICC stage distribution between patients receiving definitive versus adjuvant radio(chemo)therapy in a young HNSCC cohort.

### 4.2. Tumor Biology in Young Patients

The high oncological burden and simultaneously lower cumulative doses of classical carcinogens in young patients raise the question of heightened cellular vulnerability in this cohort—whether from impaired DNA repair [17,18], reduced carcinogen detoxification capacity [17,19], or intrinsic epigenetic susceptibility [19]. The molecular evidence, synthesized by dos Santos-Silva et al. [19] and Kaminagakura et al. [22], suggests cell cycle deregulation as a key distinguishing feature of young HNSCC. Epigenetic mechanisms may further contribute to this cell cycle disruption without requiring underlying DNA sequence change, a pathway that is currently underexplored but potentially of great relevance to young patients with limited classical risk factor exposure [20]. Histological grade presents the most heterogeneous picture across studies. Révész et al. [18] found well-differentiated tumors to be significantly more common in young patients, while Kaminagakura et al. [22] found a striking predominance of poorly differentiated tumors specifically in their young group. Santos-Silva et al. and Okuyama et al. found no significant difference in histological grade between young and older patients [15,19]. This divergence underscores that conventional histological grading alone cannot reliably characterize the biological aggressiveness of HNC in young patients.

### 4.3. Modifiable Risk Factors

A growing body of evidence highlights a distinct and increasingly prevalent subgroup of young HNSCC patients who lack classical risk factors entirely. Tran et al. [9] comprehensively reviewed the literature on non-smoking, non-drinking (NSND) oral cavity SCC and identified this subset as a distinct clinical entity. While this group appears to have similar or modestly better survival compared to patients with classical risk factor profiles [9,11], the absence of identifiable preventable risk factors makes earlier detection substantially more challenging, underscoring the need for heightened clinical awareness.

Hence, the identification of modifiable and non-modifiable risk factors is of central importance for the clinical management of young HNC patients, as these factors not only influence oncological outcomes but may also guide decisions regarding surveillance intensity and supportive care. In our multivariate analysis, nicotine abuse emerged as the strongest modifiable independent predictor of overall survival (HR 2.887; 95% CI 1.408–5.924; *p* = 0.004), alongside pre-existing comorbidities (HR 2.871; 95% CI 1.372–6.005; *p* = 0.005). This finding stands in partial contrast to the results reported by Zou et al. [4], who identified daily alcohol consumption as the dominant independent predictor of OS (HR 2.522; 95% CI 1.172–5.426; *p* = 0.018). Similarly, Frei et al. [3] found that low or absent alcohol consumption was significantly associated with improved DFS, whereas smoking did not reach statistical significance in their analysis. The divergence between our findings and those of Zou et al. and Frei et al. with respect to the dominant substance use variable may reflect differences in how substance use was defined and documented retrospectively, or random variation given the small sample sizes inherent in this field. Nevertheless, the convergence of these three studies on substance use as a key prognostic determinant underscores the importance of thorough documentation and counseling regarding both risk behaviors at the time of initial diagnosis. This aligns with further literature demonstrating a dose-dependent relationship between tobacco and alcohol exposure and survival outcomes in both young and older patients [6,9].

The prognostic relevance of pre-existing comorbidities as an independent OS predictor in our cohort deserves particular attention in the context of the young patient population. Comorbidities are typically considered less prevalent in young adults, and this assumption may paradoxically lead to underestimation of their prognostic impact. However, Piccirillo demonstrated that comorbidity carries significant independent prognostic weight in head and neck cancer patients even after adjustment for TNM stage, with moderate comorbidity associated with approximately twice the risk of death compared to patients without comorbidity [24]. This is consistent with findings from Dougherty et al. [5], who observed that among young patients with non-HPV-related HNSCC, better survival after recurrence was largely attributable to a lower Charlson Comorbidity Index (mean CCI 2.0 versus 4.8 in older controls; *p* < 0.01), which enabled more aggressive salvage approaches including repeat surgery.

### 4.4. Treatment Response and Recurrence as Prognostic Markers

The absence of complete remission at 12 weeks post-treatment emerged in our analysis as the single strongest independent predictor of both OS (HR 25.676; 95% CI 7.261–90.778; *p* < 0.001) and PFS (HR 10.139; 95% CI 3.592–28.620; *p* < 0.001). While early treatment response is broadly recognized as prognostically decisive in HNC, the magnitude of this association in our cohort is striking. Kourelis et al. [25] similarly identified regional recurrence as a particularly unfavorable event in young HNSCC patients, observing that 87.5% of young patients who developed post-treatment cervical nodal disease died within four years of initial diagnosis. The heightened consequence of treatment failure in this population reinforces the need not only for optimized primary treatment intensity but also for vigilant, protocol-driven surveillance during the post-treatment period.

### 4.5. Limitations

The median follow-up duration of 21.9 months (IQR 9.7–69.0 months) in our cohort is shorter than that reported in comparable studies. It should be noted, however, that the third quartile follow-up of 69.0 months is considerably more favorable and suggests that a substantial proportion of patients were followed over a clinically meaningful period. The short median is predominantly driven by patients referred from affiliated partner hospitals of the University Hospital Regensburg, for whom long-term follow-up data were considerably more difficult to obtain, rather than reflecting uniformly limited observation across the entire cohort. This is also reflected by the inclusion of patients treated up to 2023, leaving limited observation time for more recently treated cases. Notwithstanding, the strong prognostic impact of early treatment response and recurrence identified in our multivariate analyses is unlikely to be fundamentally altered by longer follow-up, as these events predominantly occurred within the first two years after treatment completion.

A further notable limitation of this study is the considerable heterogeneity of tumor entities included in our cohort. While the majority of patients presented with oropharyngeal (33.0%) and oral cavity (23.9%) carcinomas, our analysis additionally included nasopharyngeal carcinomas (9.1%), cancers of unknown primary (5.7%), salivary gland tumors (2.3%), and tumors of the nose and paranasal sinuses (2.3%). Oral cavity and oropharyngeal carcinoma themselves present with clinical and biological differences despite their anatomical proximity. Oral cavity carcinoma is predominantly associated with tobacco and alcohol exposure, typically requires primary surgical resection and generally carries a more favorable prognosis compared to oropharyngeal carcinoma. Louredo et al. reported 5-year OS rates of 30.9% for oral cavity cancer versus 22.6% for oropharyngeal cancer in a large population-based cohort, reflecting the substantial variability in outcomes across subsites [26]. Oropharyngeal cancer, by contrast, is increasingly HPV-driven —particularly in younger patients—and responds favorably to definitive radio(chemo)therapy, with HPV-positive tumors demonstrating markedly superior outcomes compared to HPV-negative disease.

HPV status is unavailable in a substantial proportion of our cohort. Two key factors account for this missing data. First, the study spans a period from 2002 to 2023, and HPV testing was not routinely performed in the earlier years of this interval, as it had not yet been established as a standard diagnostic procedure at that time. Second, a considerable proportion of patients were not eligible for HPV testing in routine clinical practice, as current guidelines do not recommend HPV status assessment outside of oropharyngeal carcinoma [27]. These entities differ substantially from HNSCC with respect to tumor biology, etiology, radiosensitivity, recurrence patterns, and overall prognosis. The inclusion of these entities alongside squamous cell carcinomas of the more commonly studied subsites introduces biological heterogeneity that limits the direct comparability of our survival data. Consequently, the survival outcomes reported in this study should be interpreted with caution, as they reflect a mixed-entity cohort rather than a biologically homogeneous population.

## 5. Conclusions

This retrospective multi-center analysis demonstrates that young patients with HNC face a substantial oncological burden. Early treatment response and disease recurrence emerged as the strongest independent determinants of overall survival, while nicotine abuse and pre-existing comorbidities were identified as key risk factors. From a clinical perspective, these findings may encourage clinicians to maintain a heightened awareness of early treatment response assessment and risk factor modification when managing this vulnerable patient group, while acknowledging that definitive practice-changing recommendations await confirmation in prospective studies.

## Figures and Tables

**Table 1:** Patient characteristics.

Patient Characteristics		Percentage
Patients [n]	88	
Gender [n]		
• female	24	27.3%
• male	64	72.7%
Age [years] at diagnosis		
• median	38.2	
• first quartile	35.8	
• third quartile	39.8	
Karnofsky Performance Status (KPS) prior to treatment [n]		
• 90–100%	53	60.2
• 70–80%	21	23.9
• 50–60%	1	1.1
• Not specified	13	14.8
Body Mass Index (BMI) prior to treatment [n]		
• <18.5	8	9.1
• 18.5–24.9	31	35.2
• 25.0–29.9	18	20.5
• >30.0	9	10.2
• Not specified	22	25.0
Smoking [n]		
• Smoker/former smoker	56	63.6
• Smoker with ≥30 pack years	19	21.6
• Non-smoker	23	26.1
• Not specified	9	10.2
Alcohol abuse [n]		
• Abuse/former abuse	36	40.9
• None	42	47.4
• Not specified	10	11.4
Pre-existing comorbidities		
• Yes	42	47.4
• No	37	42.0
• Not specified	9	10.2
Tumor entity [n]		
• Oropharynx	29	33.0%
• Oral cavity	21	23.9%
• Hypopharynx	9	10.2%
• Larynx	6	6.8%
• Cancer of unknown primary (CUP)	5	5.7%
• Nasopharynx	8	9.1%
• Nose and paranasal sinuses	8	9.1%
• Salivary glands	2	2.3%
UICC-classification [n]		
III	10	11.4%
IVa	57	64.8%
IVb	6	6.8%
IVc	1	1.1%
HPV status [n]		
Positive	8	9.1%
Negative	35	39.8%
Not specified	45	51.1%
Tumor follow-up [months]		
• Median	21.9	
• First quartile	9.7	
• Third quartile	69.0	
Observation period [months]		
• Median	35.4	
• First quartile	12.8	
• Third quartile	118.8	

**Table 2:** Treatment characteristics.

Treatment Characteristics		Percentage
Patients [n]	88	
Postoperative or adjuvant R(C)T	52	59.1%
Definitive R(C)T	36	40.9%
Concomitant chemotherapy [n = 87; 98.9%]	59	67.0%
Full initially prescribed radiation dose received	80	90.9%
Radiation dose [Gy]		
• Median	66	
• First quartile	60	
• Third quartile	69.6	
Boost [n]		
• Sequential	67	76.1%
• Concomitant	9	10.2%
• Without	12	13.6%
Nutrition during radiotherapy [n]		
• Oral	26	29.5%
• Parenteral or PEG tube	53	60.2%
• Not specified	9	10.2%
Acute toxicity according to CTCAE-Version 5.0 [n = 73; 83.0%]		
• Grade ≤ II	43	48.9%
• Grade ≥ III	30	34.1%
Any late toxicity according to CTCAE-Version 5.0 [n = 64; 72.7%]	40	45.5%
Weight 6 months after compared to weight before start of therapy [n = 60; 68,2%]		
• Weight loss 10.1–30.0 kg	14	15.9%
• Weight loss 5.1–10.0 kg	17	19.3%
• Weight loss 0.1–5.0 kg	19	21.6%
• Weight gain 0.0–5.0 kg	10	11.4%

Abbreviations: CTCAE (Common Terminology Criteria for Adverse Events), Gy (Gray), RCT (Radiochemotherapy), RT (Radiotherapy).

## Data Availability

The original contributions presented in the study are included in the Supplementary Materials. Further inquiries can be directed to the corresponding author.

## Supplementary Materials

The following supporting information can be downloaded at: https://www.mdpi.com/article/10.3390/medicina62071383/s1.

## Abbreviations

The following abbreviations are used in this manuscript:

CI	Confidence interval
CTCAE	Common Terminology Criteria for Adverse Events
CUP	Cancer of unknown primary
DFS	Disease-Free survival
Gy	Gray
HNC	Head and neck cancer
HNSCC	Head and neck squamous cell carcinoma
HR	Hazard-ratio
IMRT	Intensity-modulated radiotherapy
IQR	Interquartile range
OS	Overall survival
PFS	Progression-free survival
RCT	Radiochemotherapy
RT	Radiotherapy
VMAT	Volumetric modulated arc therapy
